# Comparing of the Effects of Hypericin and Synthetic Antidepressants on the Expression of Morphine-Induced Conditioned Place Preference

**Published:** 2011

**Authors:** Assad Assadi, Mohammad Reza Zarrindast, Abolghasem Jouyban, Morteza Samini

**Affiliations:** a *Science and Research Branch, Islamic Azad University (IAU), Tehran, Iran.*; b *Department of Pharmacology, Faculty of Medicine, Tehran University of Medical Sciences, Tehran, Iran.*; c *Department of Pharmaceutical and Food Control, Faculty of Pharmacy, Tabriz University of Medical Sciences, Tabriz, Iran.*; d *Department of Pharmacology, Faculty of Specialized Veterinary Science, Science and Research Branch, Islamic Azad University (IAU), Tehran, Iran.*

**Keywords:** Hypericin, Antidepressant drug classes, Morphine, Conditioned place preference, Wistar rats

## Abstract

The effect of hypericin on the expression of morphine-induced conditioned place preference (CPP) was investigated and compared with the effect of the synthetic antidepressants. The CPP paradigms took place over six days using an unbiased procedure. The results demonstrate that intra-peritoneal (IP) injection of morphine sulfate (2.5, 5 and 10 mg/Kg) significantly induce the CPP in rat. Intra-peritoneal and intracerebroventricular (ICV) injection of hypericin and/or synthetic antidepressants augmented morphine-induced CPP. It has been suggested that the adrenergic, serotonergic and dopaminergic neurotransmissions play an important role in mediating the antidepressant effect of hypericin and this effect may be due to its inhibitory effect on the reuptake of neurotransmitters. Morphine produces a reinforcement (reward) effect by activating. The *μ*-receptors that facilitate dopaminergic transmission. Serotonin is also a potent stimulator of dopamine release in such a way that an increase in brain serotonin could possibly stimulate the dopaminergic system. In conclusion, it may suggest that the augmentation of morphine-induced CPP by hypericin and synthetic antidepressants may be related to the increasing dopamine and serotonin concentrations in synaptic clefts.

## Introduction

Hypericin is one of the naphthodianthron constituents of St. John’s Wort (*Hypericum perforatum *L, SJW) belonging to the Hypericaceae family (1, 2). Clinical studies confirm that SJW extracts are effective for the treatment of mild to moderate depression and their effects are comparable to low dose tricyclic antidepressants (TCAs), but with less pronounced side effects (3). The antidepressant activity of SJW extracts has been attributed to hyperforin, hypericin and pseudohypericin and also to several flavonoids (3). From a pharmacological point of view, hypericin is the most interesting compound of H. perforatum L. (4, 5). It has been shown that the highest hypericin ratio was determined in flowers and buds generally collected between 8:00 and 10:00 AM within a day for examined hypericum populations (5). Butterweek *et al. *have suggested that adrenergic and/or serotonergic neurotransmission may be involved in antidepressant effect of TCAs and hypericin. Dopaminergic neurotransmission in the hypothalamus may also play an important role in mediating the antidepressant effects of SJW and hypericin (3). It has been shown that their antidepressant activity is related to inhibiting the reuptake of neurotransmitters such as serotonin, dopamine and norepinephrine (6). Hypericin also inhibits MAO_A_ and MAO_B_ activities *in-vitro *(7). St. John’s Wort can also cause serotonin syndrome when used in combination with the other drugs. This syndrome is the consequence of excess serotonergic activity in the central nervous system (8).

Conditioned Place Preference (CPP) is a widely-used experimental model for studying the rewarding properties of drugs in mice and rats. This animal model of drug reinforcement and drug dependence is suitable for studying the relationship between the rewarding stimulus properties of drugs and environmental stimuli. It has been demonstrated that the association of distinctive environmental stimuli with a primary reward such as food or a drug injection will result in an acquired preference for those specific environmental stimuli in the absence of the primary reward (9, 10). The majority of abused drugs, including morphine, readily condition a place preference in rodents. The production of rewarding and reinforcing effects is caused by the activation of *μ*-receptors; since reinforcement is antagonized by naloxone and the *μ*-receptor knock-out mice do not exhibit signs of morphine withdrawal (11). Disruption of morphine-induced conditioned place preference by naloxone suggests a role for opioid receptor mediation of the rewarding effects of morphine (9). The morphine-induced reinforcement effect could be due to the facilitation of dopaminergic transmission through stimulation of dopamine release (12-14). Release of dopamine from neurons in the presynaptic ventral tegmental area (VTA) into the nucleus accumbens causes reinforcement of the behavior (14). In addition to the dopaminergic system, opioidergic neurons interact with the serotonergic system. Serotonin is a potent stimulator of dopamine release (15, 16, 11). Therefore, an increase in brain serotonin could possibly stimulate the dopaminergic system (17). In this study, experiments were carried out to compare the effects of hypericin and synthetic antidepressants on the expression of morphine-induced conditioned place preference in rats.

## Experimental

*Animals*

Adult male Wistar rats (Pasteur Institute, Tehran, Iran) used in the experiments, were 220-250 g at the beginning. Animals were housed four per cage and maintained at 24 ± 0.5°C with a controlled 12 h light-dark schedule with *ad libitum *food and water except during the experimental procedures. Each treatment group consisted of eight animals. There were seven or more days between the delivery of animals and the onset of experiments. Each animal was used only once and attention was paid to the ethical principles established in accordance with the committee of ethics of the Faculty of Medicine, Tehran University of Medical Sciences.

*Methods*

**Figure 1:**
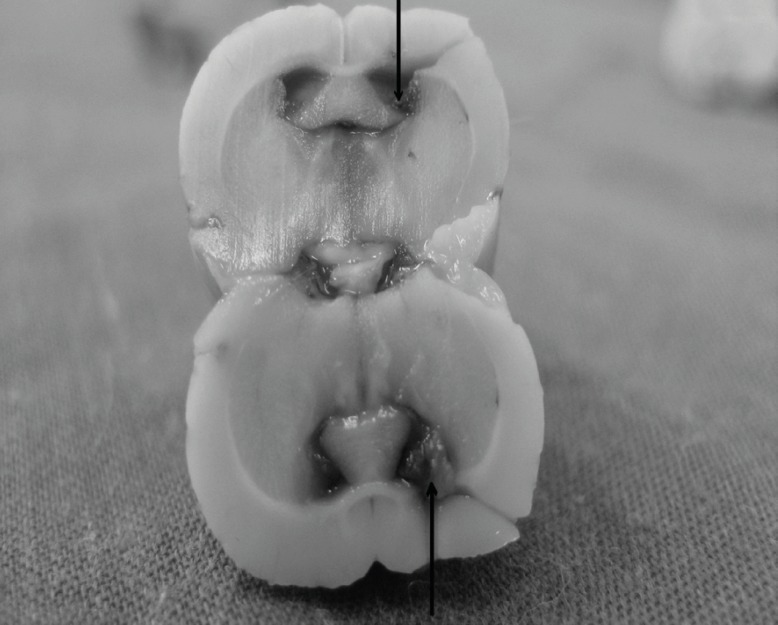
Canula placement confirmation by histological staining, the position has been pointed by a flash.

Animals were anesthetized through intra-peritoneal injections of xylazine (5 mg/Kg) and ketamine (80 mg/Kg) and placed into a stereotaxic device. An incision was made along the midline, the scalp retracted and the area surrounding bregma was cleaned and dried. In addition, lidocaine (2%) with epinephrine (0.3 mL) was injected into several locations around the incision for the purpose of local analgesia (19). A stainless steel guide cannula (22-gauge), 12 mm in length, was aimed at the left lateral ventricle (stereotaxic coordinates: 1.0 mm posterior to Bregma, 1.6 mm left lateral to midline, and 4.5 mm vertical from surface of the skull) (20). After cleaning the skull, a small amount of dental acrylic cement was pasted on to the surface so that it covered the skull screws and secured the implantation cannula in place. After the cement was completely dried and hardened, a stainless steel stylet was used to occlude the guide canula during the recovery and between drug injections. The incision was saturated and topical antibiotics were applied to the wound. The rat was removed from the stereotaxic apparatus and placed on a 37°C warming plate to allow them to recover from anesthesia. After surgery, the animals were individually housed and allowed to recover for seven days before experimental treatment. After the experiments were completed, cannula placement was confirmed by the infusion of 1% methylene blue solution (0.5 μL) and subsequent dissection. Figure 1 is confirming the canula placement.

*Materials*

The used drugs were morphine sulphate (Temad, Iran), hypericin (Tocris, USA), fluoxetine hydrochloride (Cipla, India), imipramine hydrochloride (Sobhan Daru, Iran) and tranylcypromine sulphate (Goldshield Pharmaceuticals, South Africa). All drugs, with the exception of hypericin, were dissolved in saline; hypericin was dissolved in 1% ethanol and the solutions were injected IP or ICV. Hypericin is barely soluble in water; therefore, 5 mg of hypericin was nearly dissolved in 0.5 mL ethanol and further diluted with saline to a final concentration of 0.1 mg/mL. The ethanol concentration of the hypericin solution was 1% (21, 22). During the 2^nd^ and 4^th^ days, animals were injected with saline as a control for fluoxetine hydrochloride, imipramine hydrochloride and tranylcypromine sulphate.

*Intracerebroventricular injection*

Two μL of microsyringes were used to inject the drugs. Polyethylene tubing was used to attach the injection cannula to the microsyringe and 0.5 μL of hypericin, fluoxetine, imipramine or tranylcypromine solutions were delivered slowly over a 30 sec time period.

*Apparatus procedure*

A three-compartment place preference apparatus was made of Plexiglas, measuring 88 × 36 × 34 cm, consisting of two main compartments measuring 39 × 36 × 34 cm, one having grey sides with a smooth grey floor, the other having black and white stripes (2 cm wide) with a smooth white floor. The third compartment consisted of a white central platform measuring 10 × 36 × 34 cm, which was raised by 2 cm and separated the two main compartments. During the conditioning phase, compartments were isolated using guillotine doors (23).

*Place conditioning*

An unbiased CPP paradigm was used for six continuous days and consisted of three distinct phases including: preconditioning, conditioning and postconditioning. Animals were tested during the same time period (9:00 and 14:00 h) each day for each of the CPP paradigm phases (11, 24).

*Preconditioning phase*

On the first day of the trial, each rat was placed separately in the apparatus for 10 min with free access to all compartments and the time spent in each compartment was recorded to determine the least preferred side for the animals (11, 25, 19).

*Conditioning phase*

This phase involved four days. On the first and third days, animals were injected with morphine sulphate (5 mg/Kg) and confined to their least preferred compartment (white side) for 30 min. During the second and fourth days, animals were injected with saline and confined to their preferred compartment (grey side) for 30 min (14, 23).

*Post-conditioning phase*

This phase was carried out on the 6^th^ day of the trials. Drugs (hypericin, fluoxetine, imipramine and tranylcypromine) were injected IP 15 min before or ICV 1 min before the beginning of this phase of the test (24, 11) and rats were allowed free access to all compartments for 10 min. No morphine injection was given during this phase (drug-free state). The time spent in the least preferred side (drug side) was recorded for each animal and the change in preference (CIP) was calculated as the difference (in sec) between the time spent in the drug side compartment during the post- and preconditioning phases (11, 14).

*Measurement of conditioned place preference produced by morphine*

Different doses of morphine were injected IP for the assessment of dose dependency of morphine-induced CPP.

*Induction and assessment of place conditioning by morphine sulphate*

In this experiment, the effect of morphine sulphate (2.5, 5 and 10 mg/Kg, IP) on producing place preference was tested. On the first and third days of the conditioning phase, animals received morphine and were placed in the drug side of the apparatus for 30 min. On the second and fourth days of the conditioning phase, animals received saline (1 mL/Kg, IP) and were placed in the preferred side of the apparatus for 30 min (14). It has been shown that CPP produced by morphine is dose-dependent and the submaximal and maximal responses are obtained by using 5 and 10 mg/Kg of morphine respectively (14, 24).

*Measurement of the effect of hypericin on the expression of CPP induced by morphine sulphate*

In order to test the effect of hypericin on the expression of morphine-induced CPP, this drug was injected IP (15 min) or ICV (1 min) before the postconditioning phase.

*Measurement of the effect of synthetic antidepressant on the expression of CPP induced by morphine sulphate*

In order to test the effect of fluoxetine, imipramine and tranylcypromine on the expression of morphine-induced CPP, these drugs were injected IP (15 min) or ICV (1 min) before the post-conditioning phase.

*Statistical analyses*

Values were reported as the mean change in preference ± SEM, and difference in time (sec) spent in the least preferred compartment before and after conditioning. One-way ANOVA followed by a Tukey test was used to calculate significance levels between the drugs. A value of p < 0.05 was considered significant.

## Results and Discussion

*Dose-response curve for CPP induced by morphine*

Intra-peritoneal injection of morphine sulfate (2.5, 5 and 10 mg/Kg) in rats caused a dose-dependent CPP (Table 1). We used submaximal dose (5 mg/Kg) of morphine in this experiment. The saline control group showed no preference for either of the compartments (p < 0.0001).

**Table 1:** Dose dependent morphine-induced CPP

**Phases**	**Saline (1 mL/Kg)**	**Morphine (2.5 mg/Kg)**	**Morphine (5 mg/Kg)**	**Morphine (10 mg/Kg)**
Preconditioning	209.30 ± 6.74	208.46 ± 2.26	201.77 ± 5.15	201.51 ± 4.60
Postconditioning	211.88 ± 5.84	224.05 ± 3.15	227.88 ± 5.19	238.44 ± 3.87
*Change in preference*	2.58 ± 1.07	15.59 ± 1.60***	26.10 ± 1.69***	36.92 ± 1.87***

Data are presented as mean ± SEM of eight rats. ***p < 0.0001 vs. Saline treated group of the same day. Change in preference (sec) means differences between the times spent on the drug side on post-and preconditioned phases.

*Effects of IP injections of hypericin on the expression of morphine-induced CPP*

As shown in Table 2, hypericin (25, 50 and 100 μg/Kg) increased the expression of morphine-induced (5 mg/Kg) CPP dose-dependently. The Saline and Saline + ethanol (1%) control group showed no preference for either of the compartments (p < 0.0001).

**Table 2:** The effect of IP injection of hypericin on expression of morphine-induced CPP

**Phases**	**Control**		**Morphine (5 mg/Kg)**		
	**Saline** **(1 mL/Kg)**	**Saline + Ethanol (1%) (1 mL/Kg)**	**Hypericin** **(25 μg/Kg)**	**Hypericin ** **(50 μg/Kg)**	**Hypericin** **(100 μg/Kg)**
Preconditioning	206.88 ± 2.47	208.70 ± 3.55	206.26 ± 3.30	203.42 ± 5.89	209.94 ± 4.72
Postconditioning	208.26 ± 2.31	211.98 ± 2.48	228.07 ± 2.49	235.02 ± 5.96	252.22 ± 5.69
Change in preference	1.38 ± 1.02	3.28 ± 1.28	20.56 ± 1.53***	31.60 ± 2.18***	42.27 ± 2.87***

Data are presented as mean ± SEM of eight rats^. ^^***^p < 0.0001 vs. saline and saline + ethanol (1%) treated groups of the same day. Change in preference (sec) means differences between the times spent on the drug side on post and preconditioned phases.

*Effects of IP injections of fluoxetine on the expression of morphine-induced CPP*

Fluoxetine (2.5, 5 and 10 mg/Kg) increased the expression of morphine-induced (5 mg/Kg) CPP dose-dependently. The saline control group showed no preference (p < 0.0001) for either of the compartments (Table 3).

**Table 3:** The effect of IP injection of fluoxetine on expression of morphine-induced CPP

**Phases**	**Control**	**Morphine (5 mg/Kg)**		
	**Saline (1 mL/Kg)**	**Fluoxetine (2.5 mg/Kg)**	**Fluoxetine (5 mg/Kg)**	**Fluoxetine (10 mg/Kg)**
Preconditioning	201.16 ± 11.25	198.53 ± 6.68	187.96 ± 13.24	193.44 ± 14.42
Postconditioning	203.22 ± 10.50	219.72 ± 5.49	221.94 ± 13.31	238.09 ± 12.80
*Change in preference*	2.06 ± 1.18	21.18 ± 1.61***	33.98 ± 1.19***	44.64 ± 2.81***

Data are presented as mean ± SEM of eight rats. ^***^p < 0.0001 vs. saline treated group of the same day. Change in preference (sec) means.differences between the times spent on the drug side on post-and preconditioned phases

*Effects of IP injections of imipramine on the expression of morphine-induced CPP*

Imipramine (5, 10 and 20 mg/Kg) increased the expression of morphine-induced (5 mg/Kg) CPP dose-dependently. The saline control group showed no preference (p < 0.0001) for either of the compartments (Table 4).

**Table 4:** The effect of IP injection of imipramine on expression of morphine-induced CPP

**Phases**	**Control**	**Morphine (5 mg/Kg)**		
	**Saline (1 mL/Kg)**	**Imipramine 5 mg/Kg**	**Imipramine 10 mg/Kg**	**Imipramine 20 mg/Kg**
Preconditioning	203.61 ± 5.01	221.89 ± 6.86	206.47 ± 11.37	203.63 ± 5.46
Postconditioning	207.50 ± 4.35	241.77 ± 6.74	237.31 ± 11.90	244.08 ± 4.29
*Change in preference*	3.89 ± 1.56	19.88 ± 0.66***	30.84 ± 1.84***	40.44 ± 1.46***

Data are presented as mean ± SEM of eight rats. ^***^p < 0.0001 vs. saline treated group of the same day. Change in preference (sec) means differences between the times spent on the drug side on post- and preconditioned phases.

*Effects of IP injections of tranylcypromine on the expression of morphine-induced CPP*

Tranylcypromine (5, 10 and 20 mg/Kg) increased the expression of morphine-induced (5 mg/Kg) CPP dose-dependently. The saline control group showed no preference (p < 0.0001) for either of the compartments (Table 5).

**Table 5:** The effect of IP injection of tranylcypromine on expression of morphine-induced CPP

**Phases**	**Control**	**Morphine (5 mg/Kg)**		
	**Saline ** **(1 mL/Kg)**	**Tranylcypromine ** **(5 mg/Kg)**	**Tranylcypromine ** **(10 mg/Kg)**	**Tranylcypromine ** **(20 mg/Kg)**
Preconditioning	206.02 ± 5.95	209.73 ± 8.43	207.54 ± 4.93	214.53 ± 5.24
Postconditioning	209.05 ± 5.42	234.66 ± 7.72	243.34 ± 4.54	265.52 ± 6.36
*Change in preference*	3.03 ± 0.90	24.92 ± 2.70***	35.80 ± 1.94***	50.98 ± 1.80***

Data are presented as mean ± SEM of eight rats. ^***^P < 0.0001 vs. saline treated group of the same day. Change in preference (sec) means differences between the times spent on the drug side on post- and preconditioned phases.

*Effects of ICV injections of hypericin on the expression of morphine-induced CPP*

ICV injections of hypericin (0.025, 0.05 and 0.1 μg/rat) increased the expression of morphine-induced (5 mg/Kg) CPP. The saline and saline + ethanol (1%) control group showed no preference (p < 0.0001) for either of the compartments (Table 6).

**Table 6:** The effect of ICV injection of hypericin on expression of morphine-induced CPP

**Phases**	**Control**		**Morphine (5 mg/Kg)**		
	**Saline** **(1 mL/Kg)**	**Saline + Ethanol 1%** **(1 mL/Kg)**	**Hypericin** **(0.025 μg/rat)**	**Hypericin** **(0.05 μg/rat)**	**Hypericin** **(0.1 μg/rat)**
Preconditioning	215.51 ± 3.53	216.11 ± 3.14	201.94 ± 4.79	191.62 ± 2.68	195.97 ± 5.37
Postconditioning	215.51 ± 3.47	220.64 ± 3.61	248.33 ± 5.62	265.61 ± 6.17	294.78 ± 4.49
Change in preference	1.23 ± 0.88	4.53 ± 1.57	46.39 ± 3.72***	73.99 ± 4.90***	96.46 ± 5.34***

Data are presented as mean ± SEM of eight rats. ^***^p < 0.0001 vs. saline and saline + ethanol (1%) treated groups of the same day. Change in preference (sec) means differences between the times spent on the drug side on post- and preconditioned phases.

*Effects of ICV injections of fluoxetine on the expression of morphine-induced CPP*

ICV injections of fluoxetine (2, 5 and 10 μg/rat) increased the expression of morphine-induced (5 mg/Kg) CPP. The saline control group showed no preference (p < 0.0001) for either of the compartments (Table 7).

**Table 7:** The effect of ICV injection of fluoxetine on expression of morphine-induced CPP

**Phases**	**Control**	**Morphine (5 mg/Kg)**		
	**Saline (1 mL/Kg)**	**Fluoxetine (2 μg/rat)**	**Fluoxetine (5 μg/rat)**	**Fluoxetine (10 μg/rat)**
Preconditioning	204.03 ± 11.66	196.59 ± 6.33	181.13 ± 7.86	190.28 ± 14.60
Postconditioning	203.72 ± 10.37	221.76 ± 5.06	251.71 ± 11.64	266.19 ± 11.63
*Change in preference*	-0.31 ± 1.89	25.16 ± 4.73***	70.58 ± 6.89***	75.91 ± 3.52***

Data are presented as mean ± SEM of eight rats. ^***^p < 0.0001 vs. saline treated group of the same day. Change in preference (sec) means differences between the times spent on the drug side on post- and preconditioned phases.

*Effects of ICV injections of imipramine on the expression of morphine-induced CPP*

ICV injections of imipramine (5, 10 and 20 μg/rat) increased the expression of morphine-induced (5 mg/Kg) CPP. The saline control group showed no preference (p < 0.0001) for either of the compartments (Table 8).

**Table 8:** The effect of ICV injection of imipramine on the expression of morphine-induced CPP

**Phases**	**Control**	**Morphine (5 mg/Kg)**		
	**Saline (1 mL/Kg)**	**Imipramine (5 μg/rat)**	**Imipramine (10 μg/rat)**	**Imipramine (20 μg/rat)**
Preconditioning	208.32 ± 4.98	202.44 ± 8.45	207.94 ± 6.63	203.98 ± 6.09
Postconditioning	210.41 ± 5.50	235.54 ± 9.12	253.39 ± 6.64	264.09 ± 5.52
*Change in preference*	2.09 ± 1.03	33.09 ± 0.92***	45.45 ± 1.82***	60.11 ± 2.43***

Data are presented as mean ± SEM of eight rats. ^***^p < 0.0001 vs. saline treated group of the same day. Change in preference (sec) means differences between the times spent on the drug side on post- and preconditioned phases.

*Effects of ICV injections of tranylcypromine on the expression of morphine-induced CPP*

ICV injections of tranylcypromine (5, 10 and 20 μg/rat) increased the expression of morphine-induced (5 mg/Kg) CPP. The saline control group showed no preference (p < 0.0001) for either of the compartments (Table 9).

**Table 9:** The effect of ICV injection of tranylcypromine on the expression of morphine-induced CPP.

**Phases**	**Control**	**Morphine (5 mg/Kg)**		
	**Saline (1 mL/Kg)**	**Tranylcypromine (5 μg/rat)**	**Tranylcypromine (10 μg/rat)**	**Tranylcypromine (20 μg/rat)**
Preconditioning	211.93 ± 3.40	212.61 ± 6.69	199.45 ± 3.44	200.48 ± 5.96
Postconditioning	212.79 ± 3.55	247.61 ± 5.54	247.48 ± 13.22	275.21 ± 5.71
*Change in preference*	0.86 ± 1.02	35.00 ± 2.17***	48.02 ± 11.18***	77.61 ± 3.38***

Data are presented as mean ± SEM of eight rats. ^***^p < 0.0001 vs. saline treated group of the same day. Change in preference (sec) means differences between the times spent on the drug side on post- and preconditioned phases.

In the present study, the effects of hypericin and synthetic antidepressants on the expression of morphine-induced CPP have been compared. A 2008 detailed review of 29 randomized, placebo controlled trials found that St. John’s Wort was consistently more effective than placebo and equally effective to standard antidepressants (26). It has been shown that hypericin in 0.4-2.7 mg/day given 4-6 weeks to patients with mild to moderate depression compared to other antidepressants have similar treatment responders (26). It has been suggested that hypericin inhibits the reuptake of serotonin, dopamine and norepinephrine, and is as effective as standard antidepressants but with fewer side effects (3, 6). Hypericin, like synthetic antidepressants, produces serotonin syndrome when used concurrently with other serotonin reuptake inhibitors. Recently, case reports of such events have begun to trickle in. This is a potentially serious risk and SJW should not combine with prescription antidepressants except on the specific advice of a physician (27, 28, 29). Morphine produces a reward effect, which could be due to the facilitation of dopaminergic transmission by stimulating the release of dopamine (30, 12). Serotonin is a potent stimulator of dopamine release (30, 16).

## Conclusion

In conclusion, the results of this study show that IP and ICV injections of hypericin and synthetic antidepressant produced a morphine-like effect on CPP and this effect may be related to increasing dopamine and serotonine concentration in synaptic clefts

## References

[1] Bilia AR, Gallori S, Vincieri FF (2002). St. John›s wort and depression: Efficacy, safety and tolerability-an update. Life Sci.

[2] Di Carlo G, Borrelli F, Ernst E, Izzo AA (2001). St John›s wort: prozac from the plant kingdom. Trends Pharmacol. Sci.

[3] Butterweck V, Bockers T, Korte B, Wittkowski W, Winterhoff H (2002). Long-term effects of St. John’s wort and hypericin on monoamine levels in rat hypothalamus and hippocampus. Brain Res.

[4] Patocka J (2003). The chemistry, pharmacology and toxicology of the biologically active constituents of the herb Hypericum perforatum L. J. Appl. Biomed.

[5] Kaçar O, Göksu E, Azkan M (2008). Effects of morphogenetic and diurnal variability on the hypericin content in St. John’s wort (Hypericum perforatum L.). Afr. J. Biotechnol.

[6] Butterweck V (2003). Mechanism of action of St. John’s wort in depression: What is known? CNS Drugs.

[7] Suzuki O, Katsumata Y, Oya M (1984). Inhibition of monoamine oxidase by hypericin. Planta Med.

[8] Rossi S (2005). Australian Medicines Handbook. Australian Medicines Handbook, Adelaide.

[9] Philips AG, Fibiger HC, Bozarth MA (1987). Anatomical and neurochemical substrates of drug reward determined by the conditioned place preference technique. Methods of Assessing the Reinforcing Properties of Abused Drugs.

[10] Bozarth MA, Bozarth MA (1987). Conditioned place preference: A parametric analysis using systemic heroin injections. Methods of Assessing the Reinforcing Properties of Abused Drugs.

[11] Abbasi MS, Samini M, Babapour V, Ejtemaeimehr S, Cheraghiyan S, Khayat Nouri MH (2008). Potentiation on morphine-induced conditioned place preference with concurrent use of amantadine and fluvoxamine by the intra-peritoneal and intra-cerebroventricular injection in rat. Behav. Brain Res.

[12] Johnson SW, North RA (1994). Opioids excite dopamine neurons by hyper polarization of local interneurons. J. Neurosci.

[13] Nasiraei-Moghadam S, Sahraei H, Sadooghi M, Bahadoran H (2004). The effect of oral morphine administration on development of neural tube in Wistar rats. Iranian J. Pharm. Res.

[14] Samini M, Kardan A, Mehr SE (2008). Alpha-2 agonist’s decreases expression of morphine-induced conditioned place preference. Pharmacol. Biochem. Behav.

[15] Benloucif S, Keegan MJ, Galloway MP (1993). Serotonin-facilitated dopamine release in-vivo: pharmacological characterization. J. Pharmacol. Exp. Therap.

[16] Parsons LH, Weiss F, Koob GF (1996). Serotonin 1B receptor stimulation enhances dopamine mediated reinforcement. Psychopharmacol.

[17] Pothos E, Rada P, Mark GP, Hoebel BG (1991). Dopamine micro-dialysis in the nucleus accumbens during acute and chronic morphine, naloxone-precipitated withdrawal and clonidine treatment. Brain Res.

[18] Sharifzadeh M, Rezaei H, Ghamsari MR (2003). Interactive Effects of Acute and Chronic Lithium with Dopamine Receptor Antagonists on Naloxone-Induced Jumping in Morphine-Dependent Mice. Iranian J. Pharm. Res.

[19] Aujla H, Beninger RJ (2003). Intra-accumbence protein kinase C inhibitor NPC 15437 blocks amphetamine-produced conditioned place preference in rats. Behav. Brain Res.

[20] Paxinos G, Watson C (1998). The Rat Brain in Stereotaxic Coordinates.

[21] Butterweck V, Wall A, Lieflander-Wulf U, Winterhoff H, Nahrstedt A (1997). Effects of the total extract and fractions of Hypericum perforatum in animal assays for antidepressant activity. Pharmacopsychiatry.

[22] Butterweck V, Petereit F, Winterhoff H, Nahrstedt A (1998). Solubilized hypericin and pseudohypericin from Hypericum perforatum exert antidepressant activity in the forced swimming test. Planta Med.

[23] Subhan F, Deslanddes PN, Pache DM, Sewell RDE (2000). Do antidepressants affect motivation in conditioned place preference? Eur. J. Pharmacol.

[24] Zarrindast MR, Bahreini T, Adl M (2002). Effect of imipramine on the expression and acquisition on morphine–induced conditioned place preference in mice. Pharmacol. Biochem. Behav.

[25] Cami J, Farré M (2003). Mechanism of disease, drug addiction. New Eng. J. Med.

[26] Linde K, Berner MM, Kriston L (2008). St John›s wort for major depression. Cochrane Database.

[27] DeMott K (1998). St. John›s wort tied to serotonin syndrome. Clin. Psychiatry News.

[28] Gordon JB (1998). SSRIs and St. John›s wort: Possible toxicity? Am. Family Physician.

[29] Lantz MS, Buchalter E, Giambanco V (1999). St. John›s wort and antidepressant drug interactions in the elderly. J. Geriatr. Psychiatry Neurol.

